# FEA and Machine Learning Techniques for Hidden Structure Analysis †

**DOI:** 10.3390/s21155159

**Published:** 2021-07-30

**Authors:** Xijin Shi, Sheng-Jen Hsieh, Roseli Aparecida Francelin Romero

**Affiliations:** 1 Department of Mechanical Engineering, Texas A&M University, College Station, TX 77843, USA; shixijin03@gmail.com; 2 Department of Engineering Technology, Texas A&M University, College Station, TX 77843, USA; 3 Department of Computer Science, University of São Paulo, São Paulo 13566-590, SP, Brazil; rafrance@icmc.usp.br

**Keywords:** finite element analysis, machine learning, root system architecture, non-visible bubble

## Abstract

This study focuses on investigating and predicting two hidden structures: plant root system architecture and non-visible bubbles in plexiglass. Current approaches are damaging, expensive, or time-consuming. Infrared imaging was used to study the root structure and depth of small plants and to detect the diameter and depth of bubbles in plexiglass. A finite element analysis (FEA) model was built to simulate the infrared imaging process to realize the detection and prediction given the amount of heat flux required to obtain thermal images and data. For the root system, based on a tree structure thermal profile over time derived from the FEA model, a line scan method was developed to predict root structure. The main root branches can be viewed from the detection results. Polynomial regression, support vector machine (SVM), and artificial neural network (ANN) models were designed to predict root depth. For bubble defects, three ANN models were developed to predict bubble size using temperature data generated by the FEA model. Results indicated that these models provide valid predictions. Statistical tests were applied to evaluate and compare the above predictive models. Results suggest that infrared imaging and machine learning models can be used to provide information on both hidden structures.

## 1. Introduction

The hidden structure in this study is twofold: one is the root system, the other one is the non-visible bubble. The term root system architecture (RSA) refers to the combination of a plant’s roots and their multiplex components in the growing environment [1]. A robust RSA is essential for plant growth because its roots absorb water, nutrients, and other resources, dissolve ions and synthesize and store organic matter. Since water and nutrients are unevenly distributed in soil, the roots’ ability to absorb and transport significantly determines the amount of resources that plants can obtain [2]. Meanwhile, coexistence with other organisms and changes in the rhizosphere require RSA participation. According to recent research, RSA is sensitive to the availability and distribution of nutrients. Therefore, root quality can affect the productivity of crop plants [3,4]. Moreover, through physically supporting and recruiting beneficial microorganisms, RSA significantly influences microbial communities and plant performance [5]. Accordingly, the root system is fundamentally essential for plant growth, production, and survival. The investigation, analysis, and prediction of RSA can help growers create better plant growth and multiplication conditions.

Several non-destructive imaging techniques have been applied to study the spatial distribution of RSA, such as ground-penetrating radar (GPR), nuclear magnetic resonance (NMR), and computed tomography (CT). A GPR technique has been applied to study the three-dimensional distribution of large root systems and estimate the diameter of tree root systems [6,7]. In contrast, the NMR medical imaging research system showed better results for detecting tiny roots, which could investigate RSA in various soil media at a 0.6 mm resolution [8]. Moreover, Dusschoten et al. used NMR and toolbox NMRooting to monitor the root mass, length, diameter, tip number, growth angles, and spatial distribution non-destructively. More specifically, this technology can quantitatively measure tiny roots with a diameter that varies between 0.2 and 0.3 mm [9]. Nonetheless, current research applications still have limitations in terms of accuracy, application range, and cost [10]. Therefore, infrared imaging, which is an economical and non-destructive technology, has been taken into consideration. Near-infrared reflectance (NIR) has been employed to gather quantitative seed phenotypes to improve the study of seed traits [11]. It was also used to develop a fixed-point observation system that could accurately predict leaf area index [12]. Moreover, a Fourier transform infrared imaging (FTIRI) approach was developed to monitor the nutrient changes in the rhizosphere, which helped comprehend nutrient flow processes in assorted complicated biological systems [13]. Although current research on utilizing IR in the agricultural sector has achieved convincing results, applications for detecting RSA are limited. In addition, FEA can simulate the heat transfer process and provide valid results regarding various thermophysical properties and boundary conditions [14]. Therefore, in this study, it is expected that IR imaging with FEA simulation can be used to investigate and identify RSA precisely and efficiently.

The second object of this study is to detect non-visible bubbles in plexiglass boards. Plexiglass refers to poly (methyl methacrylate) PMMA, which is lightweight, has good tensile strength, UV resistance, and other outstanding properties. It is a commonly used transparent thermoplastic in various industrial fields, such as architecture and electronics [15]. The defects in the plexiglass strongly affect the products’ quality, which could result in misadventure. Additionally, defective products can lead to recall or rejection by the consumer. Hence, an accurate inspection process is necessary to ensure the plexiglass products’ high quality. Typical defects include, but are not limited to, the following types: scratch, crack, pits, and bubble. Traditionally, human vision examination has been widely used in this sector, but this work could damage inspectors’ eyes, and the accuracy depends on the inspectors’ proficiency [16]. As the demand for products increases, the requirements for inspection’s accuracy and efficiency increase as well. Hence, modern technologies have been implemented, such as machine vision, ultrasonic and X-ray. Machine vision is an approach that can automatically recognize objects based on image processing algorithms [17]. Ding et al. proposed a method to automatically detect dispersed defects in the resin eyeglass by applying machine vision methodologies. The approach was validated by simulation and experiment, which achieved a 97.50% accuracy and only took 0.636 s [18]. Machine vision algorithms have also been introduced to develop a defect detection system to inspect mobile phone screen glass, which could meet the requirements of online detection [19]. Ultrasonic has been utilized to inspect thin-walled polymer pipes, which proved that this method could detect defects down to a size of 1 mm [20]. Hassen et al. compared the performance of ultrasonic C-scan and X-ray computed tomography in detecting glass fiber and polypropylene composites with artificial defects. The results demonstrated that X-ray could identify the size and shape of defects but not work in materials with similar densities. Ultrasonic could not locate all embedded defects, but it could detect the defects’ size and shape in glass fiber composites [21]. However, current approaches are limited to cost, difficulty in operation, type, and size of defects.

Thermography has also been applied to detect materials’ defects. Pulse phase thermography could detect artificial defects with 5–6 mm depth in PMMA specimens [22]. The artificial flat-bottomed hole in PMMA samples was also studied by utilizing lock-in and pulse phase IR thermography. The experiments suggested that this method could detect a 10-mm-diameter and 3.6-mm-depth hole with an uncertainty of 17% [23]. Image processing algorithms have been implemented in this sector. Grys presented a method, including background removal, image segmentation, and feature extraction, to detect holes in the PMMA slab using active IR thermography [24]. After achieving such convincing results in the defect detection field by thermography, it is expected that IR can be successfully applied to detect non-visible bubble defects in plexiglass.

This study aims to develop an efficient, accurate, and economical approach to investigate the root system in situ and non-visible bubble defects in plexiglass by implementing thermography methodologies. More precisely, the objectives of this research are twofold. The first is to develop a new methodology to investigate the RSA and bubble based on the infrared (IR) imaging technology and evaluate the feasibility with the finite element analysis (FEA) theory. The second is to design predictive models for the information of root (structure and depth) and bubble (diameter and depth) based on FEA findings.

## 2. Implementation of FEA Theory for RSA

### 2.1. RSA Model and FEA Set-Ups

**Figure 1:**
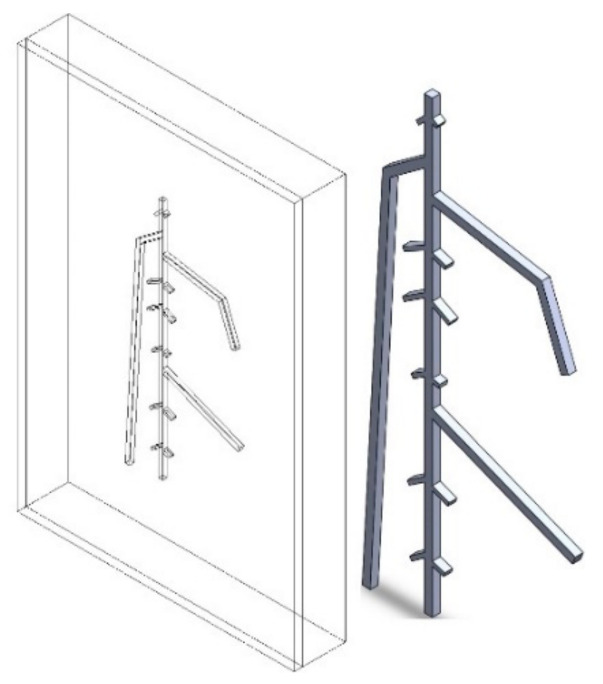
Transparent isometric view of RSA model (**left**) and isometric view of sugar beet root model (**right**).

There are two main types of root structures: taproot and fibrous root [25]. For taproot, the most central and stout part is the primary root, which grows vertically downwards. The secondary roots are tiny and spread laterally around the primary root. In contrast, the fibrous root is composed of a moderate number of small roots. In this study, sugar beet roots that have a taproot structure were investigated and modeled. The 3D model for the entire simulated sample was built as shown in Figure 1, which has three parts arranged in order from front to back as follows: acrylic glass cover, soil, and the sugar beet root system in situ.

The dimension of this sample is 65 mm × 95 mm × 12 mm. The front transparent plate is a 2-mm-thick transparent acrylic glass cover used to protect the root and soil sample. The cover also increases the emission rate, which contributes to better thermal imaging results. To simulate the root growth environment, the material of the medium was set as soil. The 1-mm-thick model of the sugar beet root is on the right. The diameters are 1 mm for the primary root and 0.5 mm for the secondary roots. The depth of the RSA was considered as the distance between the bottom surface of the acrylic glass cover and the upper surface of the root. RSA models for nine different depths were established and analyzed: 1 mm, 2 mm, 3 mm, 4 mm, 4.5 mm, 5 mm, 6 mm, 7 mm, and 8 mm. A model with a depth of 9 mm was not built due to limitations in the container’s size.

To investigate infrared imaging performance based on this RSA model, ANSYS and finite element analysis (FEA) methodologies were employed. To simplify and accelerate the simulation process, the temperature of the soil, roots, and acrylic glass was assumed to be a constant temperature of 23 °C (296.15 K) initially. As shown in Table 1, the density (ρ), thermal conductivity (k), and specific heat capacity (cp) of these three materials were given. The specific heat capacity of the roots is twice that of the acrylic glass cover, and the specific heat capacity of the acrylic glass cover is twice that of the soil. Therefore, the heating and cooling rate of the roots are lower than that of soil and acrylic glass cover, which will eventually lead to temperature differences between the root region and soil region on the cover’s surface.

To create thermal excitation inside the sample, a constant heat flux was applied to the upper surface of the acrylic glass cover for 10 s. Then the whole model was naturally cooled for 110 s. The upper surface was set under natural convection, while other outer surfaces were set as perfectly insulated to prevent heat loss. The amount of heat flux was set as 0.0005 W/mm^2^, which transmitted a total of 30.875 J energy to the surface. In experimental conditions, the infrared imaging camera would be placed directly above the object. Therefore, during the whole process, including heating and cooling stages, the data and images of the upper surface were recorded for further analysis.

**Figure 2:**
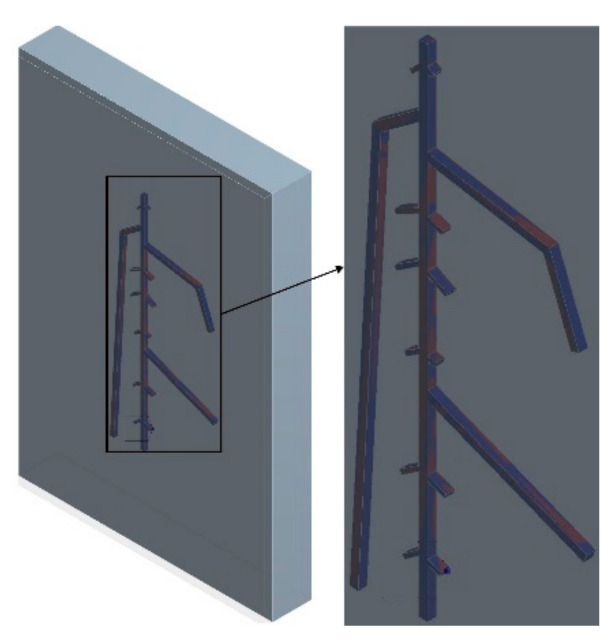
Meshed RSA model showing Node Merge contact region.

The RSA model was meshed before the initiation of the numerical simulation. The element size and method are essential factors influencing the accuracy of results during the meshing process. While an oversubtle grid can result in the unacceptable consumption of calculation resources, an overly coarse one can lead to inaccurate results. Therefore, the element size was set to 1 mm to balance these two aspects, and the meshed model is shown in Figure 2. The process generated 80,237 nodes and 53,547 elements for each model. In addition, because the contact relationship between the roots and the soil is not entirely rigid, ‘Node Merge’ was applied to the contact region, which merged a total of 2270 nodes.

### 2.2. Outcomes and Analysis

**Figure 3:**
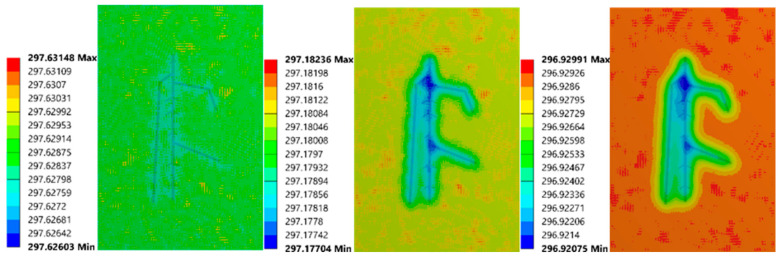
Upper surface’ thermal images of 2 mm root depth model at 17 s (**left**), 27 s (**mid**), and 40 s (**right**).

The 2 mm depth model is presented here as an example to elaborate on observations from the FEA process. Figure 3 reflects the temperature distribution on the upper surface at 17 s, 27 s, 40 s. To improve the visibility of the results, the structure of the roots was superimposed over each image. Throughout the entire process, the shape of the roots changed from blurred to clear, then to blurred. This phenomenon suggests that the temperature difference between the root and soil areas becomes bigger first, then smaller. The structure of the roots could first be viewed clearly at 17 s. At 27 s, the observed structure contained more components, but some areas were fused. At 40 s, the suggested root region was well beyond the original size of the roots. In addition, some parts of the upper surface exhibited a faster cooling rate. The regions corresponding to these sections were the joints of primary roots, which had a larger volume, which could result in an enormous temperature difference. It can be inferred that if a region has a significant temperature difference, there should be roots buried underneath. In addition, the thermal profile of the secondary roots was influenced by the primary root, while the primary root shifted the temperature difference area to its location.

**Figure 4:**
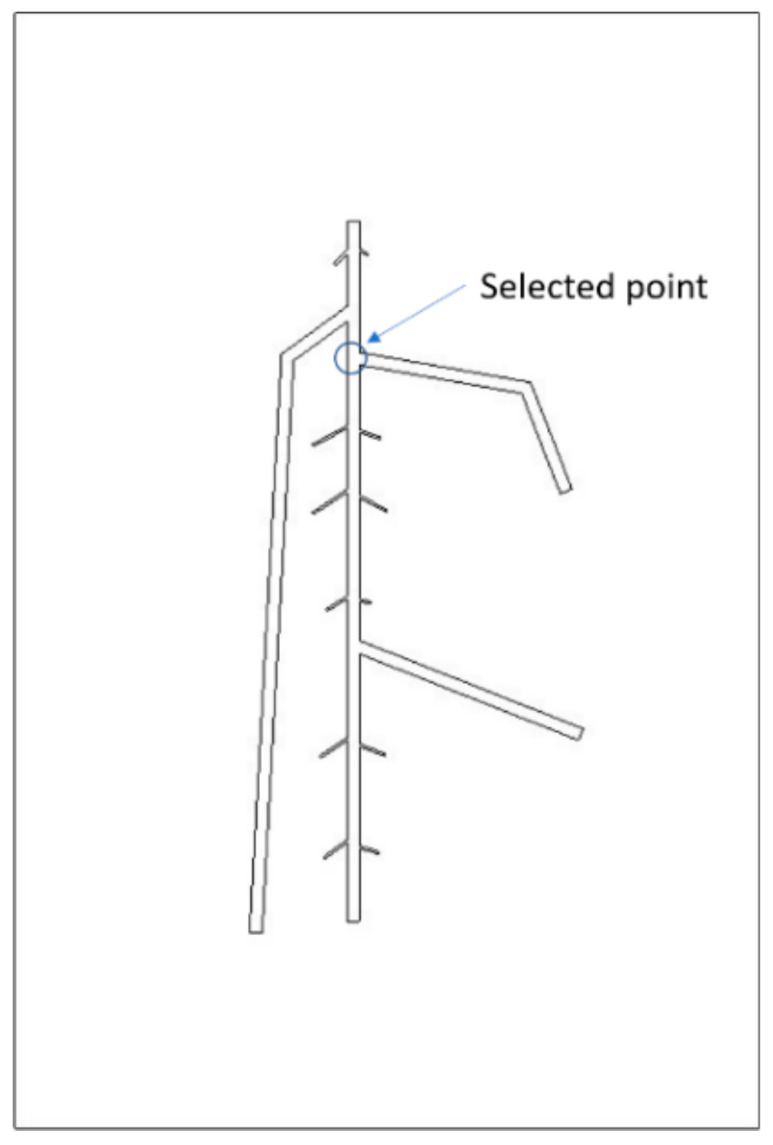
Feature point of the root.

The analysis of FEA results suggested that the primary root joint areas have more obvious temperature differences than other areas. Thus, a point from this position (shown in Figure 4) was selected for further inspection of potential relationships between root depth and temperature distribution on the upper surface.

**Figure 5:**
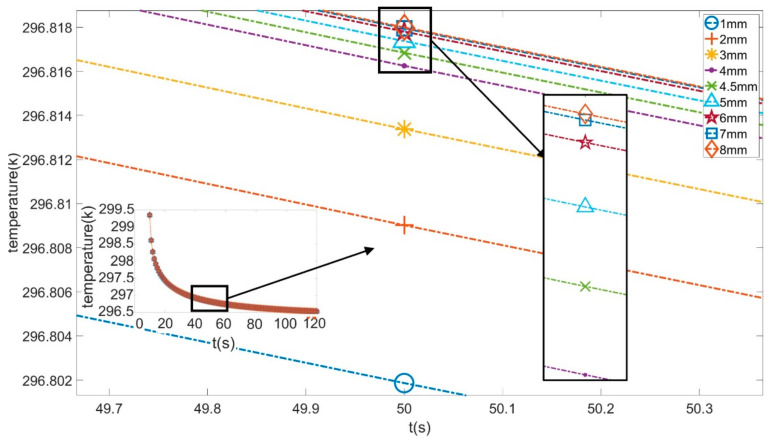
Temperature graph of the feature point during the cooling process.

The temperature graphs (around 50 s) of this point from 9 different depth models are shown in Figure 5 below.

The average temperature and cooling rate of this point’s temperature from these models are given in Table 2 below.

According to these figures and tables, the average temperature of the 1 mm depth model’s selected point during the cooling process was the lowest. As the depth increased, the average temperature kept increasing. This phenomenon was due to the closer distance between the shallowly buried root and the upper surface, which made it easier for heat energy to be released into the air; in other words, the energy gained from the heating stage and then stored at that depth outflowed faster. At deeper depths, the roots are further from the air. Thicker soil obscures the effect of the roots on the upper surface temperature distribution. As a result, a model with a deeply buried root has a lower average temperature than a shallowly buried one.

From Table 2, it can be observed that as depth increased, the cooling rate did not decrease all the time. The relationship between root depth and temperature change trend did not present a global linear property. The selected point was chosen as an example, and polynomial fitting was used to fit its cooling process. The polynomial functions and their corresponding R-squared values at three different depths are given as Equations (1)–(3). In addition, for all models, the cooling process was non-linear and thus could not be fit using linear functions.

2 mm depth model:

(1) $${y=9}^{-11}x^{6}-{\;3}^{-8}x^{5}{+5}^{-6}x^{4}-\;{0.0004x}^{3}+{0.0134x}^{2}\;0.263x+{299.11,\;R}^{2}=0.98$$

4.5 mm depth model:

(2) $${y=9}^{-11}x^{6}-{\;3}^{-8}x^{5}{+5}^{-6}x^{4}-\;{0.0004x}^{3}+{0.0134x}^{2}-\;0.2634x+{299.11,\;R}^{2}=0.9798$$

7 mm depth model:

(3) $${y=9}^{-11}x^{6}-{\;3}^{-8}x^{5}{+5}^{-6}x^{4}-\;{0.0004x}^{3}+{0.0134x}^{2}-\;0.2633x+{299.11,\;R}^{2}=0.9799$$

### 2.3. The Impact of Boundary Conditions and Front Cover

In this FEA simulation, several boundary conditions and structures of the 3D model hold the potential to affect the result. One is through heating, and the other is through the front cover. With the increase in heating power, the specimen could receive more heating energy. Therefore, the differences between hidden structures and mediums may result in a more obvious surface temperature distribution. For the front cover, a thick one may prevent internal differences from spreading to the surface. More specifically, to investigate these factors’ effects, the model with roots buried at 2 mm depth was chosen as the specimen, while the amount of heat flux, length of heating, and thickness of the front acrylic glass cover were adjusted.

**Figure 6:**
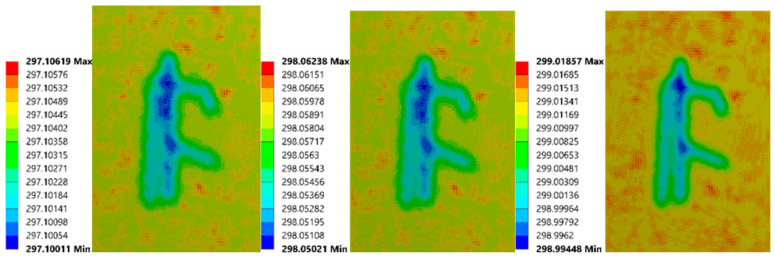
Simulation images of FEA model with heat flux of 0.0005 W/mm^2^, 0.0010 W/mm^2^, 0.0015 W/mm^2^ (from left to right).

The first parameter to be discussed is the amount of heat flux. It was set as 0.0005 W/mm^2^ in the previous simulation, then it was improved to 0.0010 W/mm^2^ and 0.0015 W/mm^2^, while the heating time remained as 10 s. The upper surface’s temperature distribution with each setting at 30 s is listed in Figure 6. From these figures, as the heating power increased, the temperature increased, but the relative temperature distribution did not change much.

**Figure 7:**
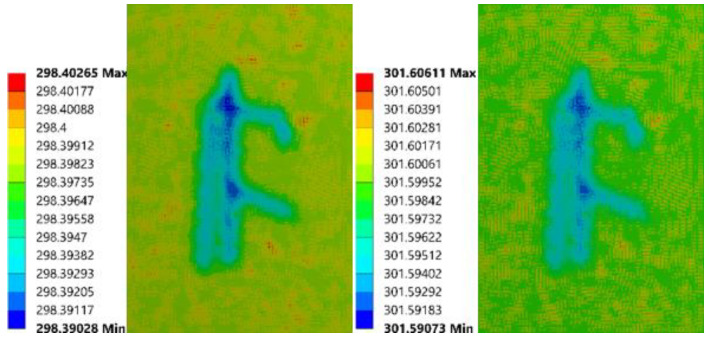
Simulation images of FEA model with 20 s (**left**) and 30 s (**right**) heating time.

The second parameter to be adjusted is the length of heating. It has been extended from 10 s to 20 s then to 30 s, while the amount of heat flux remained as 0.0005 W/mm^2^. Compared to Figure 6, the relative thermal images at 30 s in Figure 7 did not change significantly with extended heating time. The above phenomena indicate that the extension of the heating period and the improvement in heating power do not significantly contribute to inspection capability in this study.

**Figure 8:**
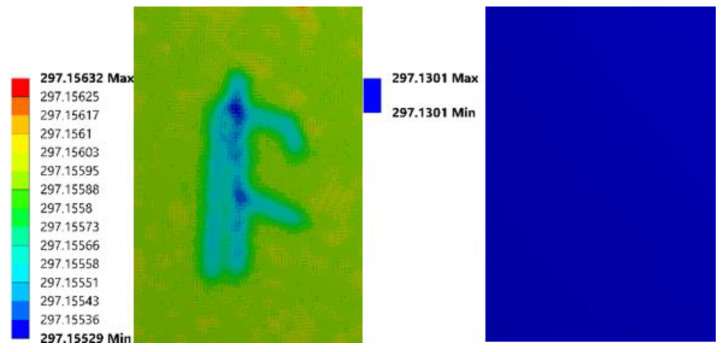
Simulation images of FEA model with 3 mm (**left**) and 4 mm (**right**) thick cover.

The last argument is the thickness of the front acrylic glass boards. It was changed to 3 mm and 4 mm, and the simulation results are given in Figure 8. The amount of heat flux was set as 0.0005 W/mm^2^, and the length of heating was 10 s. As the cover’s thickness increased, the temperature differences disappeared first, which means that the cover thickness has a significant impact on the investigation ability. Therefore, a thinner cover (≤3 mm) has the possibility to produce more valuable results. The 2 mm thick acrylic glass cover was chosen in this study because it is convenient to acquire and process.

### 2.4. Detection of the Root Structure

In order to detect the shape of the RSA, a data processing method based on differences in thermal properties between root and soil was developed. The temperature data, which contained the temperature and positions of each point on the upper surface at each time interval, were exported from ANSYS. The preliminary processing of unsorted data was finding out the type and value of all X and Y coordinates. Next, the data were sorted based on each X and Y coordinates in ascending order. Then the data scanning was done through each X and Y as the following steps.

First, start from the minimal one of the X coordinates, calculate the average temperature of each point on this line. Next, smooth the data of this line by ‘Smoothdata’ and ‘Movmean’, which can eliminate the noise, smoothen the data, and reduce the impact of irrelevant factors. Then all the minimal local value of this line is found through differences and sign functions as follows. Calculate the differences between adjacent elements of each line. Moreover, apply the sign function to convert the result into 1, 0, and −1. Then calculate the differences between adjacent numbers again. If the value is larger than 0, this would be the position of a minimal local value. Add 1 to all the founded positions, which would equal to the corresponding indexes. Export all the indexes found in this line, which are the corresponding coordinates of minimal local values. Furthermore, repeat the above steps to find the coordinates of valleys from the second smallest X coordinate to the largest one. After finishing X-Scan, redo these previous actions for each Y coordinate. Finally, combine and plot the results of the selected coordinates from the X-scan and Y-scan.

The process of the line scan method is given below as Algorithm 1:
**Algorithm 1**.Input: Temperature image at a timestamp **S_i_** = (**x_1_**, **y_1_**), …, (**x_m_**, **y_n_**) Temperature of a point (**x**, **y**) from above image **T_i_**(**x**, **y**) Timestamp **i** = **1**, …, **t** (Cooling process) Goal: Find the coordinates set of the root region **R**, Plot the image of RSA Initialize: **R** = ∅, **R_x_** = ∅, **R_y_** = ∅ For **α** = **1**, …, **m** For **β** = **1**, …, **n** **T**(**x_α_**,**y_β_**) = mean(**T_1_**(**xα**,**y_β_**), …, **T_t_**(**x_α_**,**y_β_**)) Smooth data of **T**(**x_α_**,**y_β_**) **R_α_** = (**x_α_**,**y_index1_**), **y_index1_** = position of **local minimums** **R_x_** = **R_x_**∪**R_α_** Repeat above steps to get **R_β_** = (**x_index2_**,**y_β_**) **R_y_** = **R_y_**∪**R_β_** **R** = **R_x_**∪**R_y_** Plot based on the coordinates of **R** Output: **R**, Plot of RSA

Moreover, the accuracy of this method has to be evaluated. Because the detection result produced from the previous line scan method was a binary image, the original root image was converted to a black and white image as well. Next, the original root image’s grayscale value was reduced to ten percent, while that of the predicted root image was reduced to ninety percent. Then both images with adjusted grayscale values were added together. The FEA model with roots buried at 2 mm depth is given as an example. The temperature data were imported into the line scan method, and the detected root structure is presented and evaluated.

**Figure 9:**
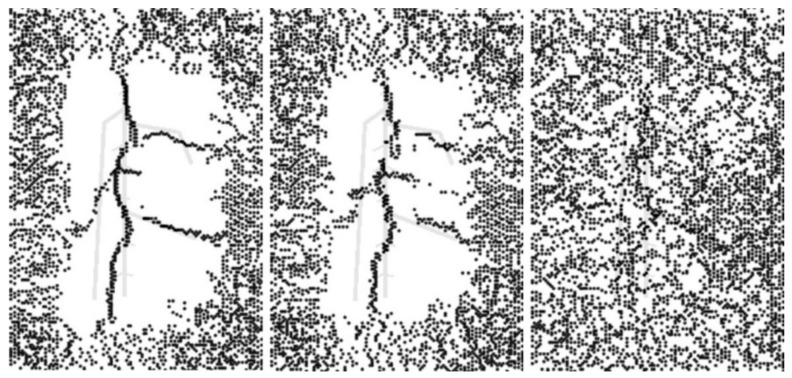
Prediction results of FEA model with roots buried at 2 mm (**left**), 5 mm (**middle**), and 6 mm (**right**).

As shown in Figure 9, the main central branch could be viewed, but it swung left and right from the original position because of the other three primary branches and secondary branches. The upper right big root position was founded, but it has deviations due to the main central branch’s existence. In contrast, since the bottom right primary root is relatively far away from other big roots, it was depicted precisely. The left primary root was almost missed because it is close to the main central branch. However, the influence of secondary roots was covered by the impact of primary roots, small roots were not located. Besides that, due to the sensitivity to subtle noise, many random noise points appear around the founded RSA structure. For the result of the 5 mm depth model, several main branches could still be detected. However, for the 6 mm depth model, root structures were not located. Therefore, the line scan method could detect the current FEA model’s root structure with a root depth that is not deeper than 5 mm.

### 2.5. Prediction of the Root Depth

#### 2.5.1. Polynomial Regression Model

A polynomial regression model is a flexible tool for revealing complex relationships between inputs and outputs. Additionally, this model allows complete control because the exponential parameters need to be set at the beginning. A certain amount of data are required to determine the appropriate exponential parameter to avoid overfitting and maintain accuracy. A polynomial regression model with n inputs is shown as Equation (4) below:

(4) $$y=\hat{\;y}{+e,\hat{\;y}=a}_{1\;}{\times \;x}_{1}{+a}_{2}{\;\times \;x}_{2}+\cdots {+a}_{n\;}{\times \;x}_{n}$$

In the above equation, x is the input, and a is the corresponding coefficient parameter, e is the constant error term, $\hat{y}$ is the estimated output, and y is the final output.

**Figure 10:**
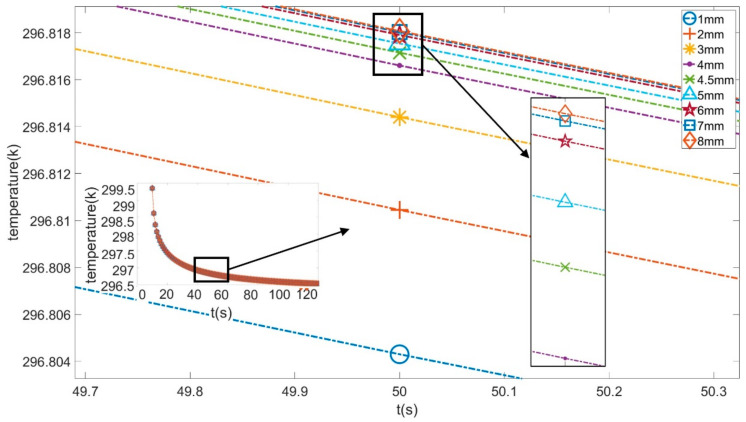
Average temperature of upper surface during the cooling process.

The first thing to do is to select the input data for building the model. The average temperature of the upper surface during all models’ cooling processes is depicted in Figure 10. As root depth increased, the average temperature of the upper surface kept increasing. Based on this phenomenon, the average temperature was then considered as the source of the input data for the polynomial regression model.

To further study the relation between root depths and the average temperature of the upper surface, the correlation coefficient (CC) was introduced. As listed in Table 3, the correlation coefficient kept increasing as time increased. Therefore, the average temperature data, ranging from 60 s to 120 s for all models, were selected as inputs, and their corresponding root depths were set as outputs to be calculated and predicted.

Two models were created: one is the interpolation model, the other one is the extrapolation model. The first interpolation model used all models except the target as inputs to predict the target (2 mm, or 3 mm, or 4 mm, or 4.5 mm, or 5 mm, or 6 mm, or 7 mm). The second extrapolation model used all models except the target to predict the shallowest one (1 mm) or deepest one (8 mm). For the first model, the average error was 1.03 mm, and the average accuracy was 68.96%. For the second approach, the average error was 2.19 mm, and the average accuracy was −104.37%. The extrapolation model established the foundation for data and provided space for upward or downward growth. Comparatively, the interpolation model sets the lower and upper limits of the data. So, the interpolation model produced more accurate results than the extrapolation model. The interpolation model to predict 4.5 mm and the extrapolation model to predict 8 mm are given below as examples.

Interpolation model to predict 4.5 mm:

(5) $$\begin{array}{l} y=-\;67777.57173641\;\times \;x_{1}+298234.7428461\;\times \;x_{2}\\ -\;404202.16199545\;\times \;x_{3}+74860.79648985\;\times \;x_{4}+72863.90284235\;\times \;x_{5}+207529.91694065\;\times \;x_{6}\\ -{186166.48290804\;\times \;x}_{7}+1380601.18953825 \end{array}$$

Extrapolation model to predict 8 mm:

(6) $$\begin{array}{l} y={54609.30507467\;\times \;x}_{1}-\;{249530.3871525\;\times \;x}_{2}+{390530.77926045\;\times \;x}_{3}\\ -\;{176754.24016523\;\times \;x}_{4}+{3332.2624362\;\times \;x}_{5}\\ -{160075.45127699\;\times \;x}_{6}+{145232.4524183\;\times \;x}_{7}-\;2177681.87002655 \end{array}$$

In the above equations, x_1_, x_2_, x_3_ x_4_, x_5_, x_6,_ x_7_ are the temperatures at 60 s, 70 s, 80 s, 90 s, 100 s, 110 s, 120 s of the model, respectively, and y is the corresponding predicted root depth.

#### 2.5.2. Data Pre-Processing for Machine Learning Models

**Figure 11:**
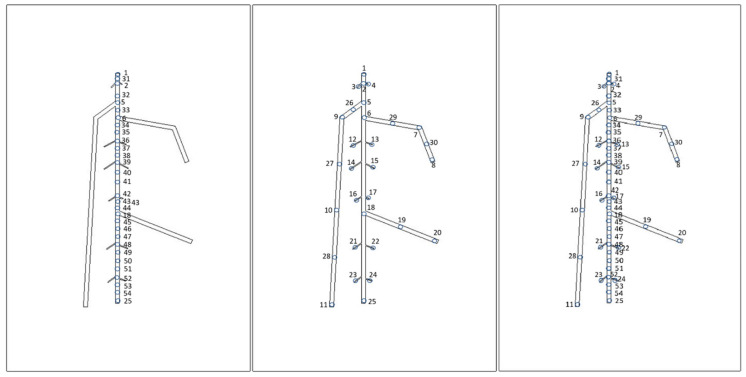
Different sets of temperature data input set 1 (**left**), input set 2 (**mid**) and input set 3 (**right**).

Points on the upper surface from different regions of the root were selected, and their temperature data over time were recorded. To compare the impact of different combinations of points on prediction performance, the points selected were from the areas corresponding to the primary and secondary roots. These points were then combined into three different sets for use as input nodes. The first set consisted of thirty points from the area corresponding to the central primary root. The second set consisted of thirty points from areas throughout the RSA. The last set was a combination of the first two. Since the points selected by the first and second overlapped, the last set has a total of 54 points. The locations of these points are shown in Figure 11. For each point, temperature data from the cooling process were extracted every three seconds. Therefore 34 sets of data of each point, considered to be 34 features, were produced. As nine different root depth models were simulated, for the input set 1 and 2, the total number of samples was 270. For the input set 3, the total was 486. The output was the corresponding root depth of each input data set.

#### 2.5.3. Support Vector Machine

To implement the multiclass classification, the LIBSVM toolbox was used, which is based on a one-to-one method. In addition, the RBF kernel (Gaussian kernel) was selected to map the low-dimensional feature space into high-dimensional feature space. Additionally, SVM has two essential factors: one is the cost (c), the other is g (gamma). Cost means tolerance of error, which determines the generalizability of the model. Gamma is a parameter of the RBF kernel, which is inversely proportional to the number of support vectors that affect training and prediction speed. Accordingly, to train an efficient model that will neither overfit nor underfit, the values of c and g must be kept within an appropriate range. Hence, grid-search and cross-validation (cv) are utilized to find the best c and g automatically. To initiate the grid-search, a set of c and g is designated for the parameters. Next, based on the selected scoring standards, the best setting would be found out after exhausting all the various combinations of parameters. To avoid the model becoming too complicated, which may lead to overfitting, cross-validation is implemented simultaneously. The training sets will be divided into several subsets randomly. One subset is chosen as a training set for each round, and others are set as validation sets. These two mechanisms were combined to adjust the parameters, which improve the training efficiency and model performance.

Since SVM is a classification technique, if the classification result is not the same as the target, the classification is considered incorrect. The results of SVM models with different input sets are given in Table 4. It can be observed that although the second input set performed well on the training data set, it had the worst accuracy on the validation set, suggesting that overfitting occurred. The third input set performed best on the validation set and had the smallest difference in accuracy between training and validation sets. Therefore, the SVM model trained by the third input set achieved the best performance.

#### 2.5.4. Artificial Neural Network

ANN models trained with a small number of samples tend to have weak generalizability and are prone to overfitting. Since the number of samples from the FEA was insufficient to train a strong ANN, K-Fold cross-validation was used to improve the utilization efficiency of the current samples. The K-fold cv contains the following four steps. The first is dividing the training sets into k groups randomly. The second is taking each unique group as a testing set and taking the other remaining groups as the training sets. A parameter is set as the evaluation standard and recorded during this process for each model. The third is comparing the parameter for all models and then picking the best one. Therefore, the designated parameter for evaluation criteria has a direct impact on the performance of the model. R squared is selected as the evaluation standard, which only varies between 0 and 1 for any different models. Intuitively, the closer R squared approaches 1, the better the model fits the data. On the contrary, if R squared is close to 0, the model almost does not fit the data. After each comparison, the model with a larger R squared is retained. Meanwhile, all models in this study were designed to have a single hidden layer. Moreover, the number of neurons in the hidden layer is a vital parameter that affects ANN performance. If the number is insufficient, the network may not actually be trained or may have poor performance. If the number is too large, although the system error can be reduced, the training time will be extended and potentially stick in a local optimum, which would cause overfitting. In consideration of this possibility, during each cross-validation, hidden layers with different numbers of neurons (ranging from 2 to 33) were compared to determine the best number. For the last step of K-fold cv, the best-performing model and the number of neurons are selected. Since K-fold cv reuses training sets to dig out the parts with the most visible features, it consumes much more computing resources than the regular ANN model. Hence, multi-core parallel operations and GPU acceleration are utilized to exploit more computing resources and then accelerate the training process.

The temperature data of each point from the cooling process were extracted every three seconds. Therefore, 34 sets of data of each point were produced, considered as 34 input nodes. To verify the effectiveness of the K-Fold cross-validation, the ANN was trained without it at first; 2/3 of the data were used as the training set; 1/3 were used as the validation set. The two parts were separated randomly to avoid overfitting and improve generalizability. The ‘Trainrp’ and ‘Mean Square Error’ training functions, which have a fast convergence rate and small memory usage, were selected. The number of neurons in the hidden layer was set as 10. As shown in Table 5, the data set of 30 points on the central primary root provided the best result.

K-fold cross-validation was then implemented to determine the optimal number of neurons in the hidden layer and improve data utilization. Again, 2/3 of the data were used as the training set for K-fold cross-validation, and 1/3 were used as external data for validation. K-fold cv separated each input set into k copies for training and validation. After comparison, the training set with the highest validation accuracy was determined as the best input data.

From the results in Table 5, the difference in accuracy between the input sets was slight. The third input set provided the best validation result, which was different from the result of the original ANN. In addition, the accuracies of all input sets were higher than their corresponding sets in the ANN models without K-fold cv. The results indicate that the use of K-fold cv to select the optimal number of neurons and training data for each input set helped to improve the performance of the ANN models.

Compared with the original ANN, the K-fold cross-validation optimized the data fitting effects and reduced error rates significantly. Nevertheless, this method reused the data multiple times, and the calculation consumption time increased dramatically. After using parallel computing and graphics card acceleration technologies, it took about half an hour to train one ANN model, which may still provide a relatively efficient approach.

Three models have been trained and tested for the prediction of root depth. The polynomial regression model used the average temperature of the upper surface during the cooling process as input, which is more convenient to acquire. Additionally, it presented the highest training speed, and the average accuracy was 68.96%. However, it could only predict one root depth at a time, while SVM and ANN could predict all root depths. SVM and ANN required appropriate input data and more time to be trained. ANN with k-fold cv produced the best accuracy while SVM consumed less time to be trained.

#### 2.5.5. Model Testing

**Figure 12:**
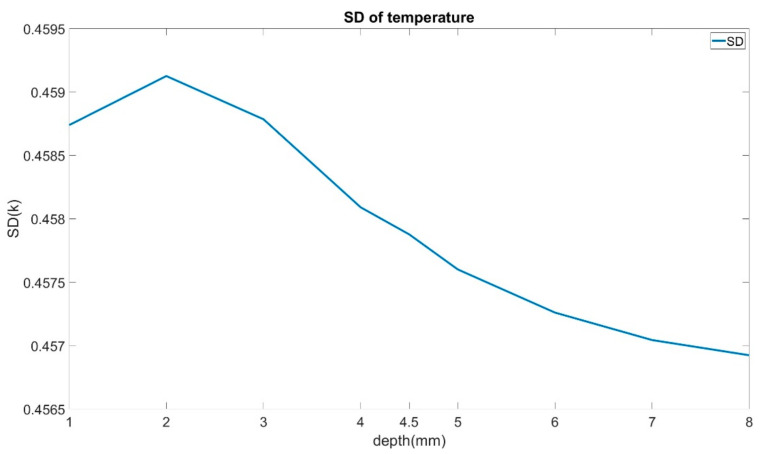
Standard deviation of all models’ temperature data.

To analyze and compare the temperature data characteristics of different depth models, standard deviation (SD) was used. Since the root depth prediction models need to be fault-tolerant, SD was also increased (in increments of 0.02) to simulate random noise due to environmental factors. Increments of 0.02 were chosen because the temperature differences between depths are relatively minimal. Figure 12 shows that SD increases from the 1 mm depth model to the 2 mm depth model, then decreases as depth increases. Although the relationship between the depth and temperature data is non-linear, the temperature at different depths exhibits minimal differences.

Statistical testing based on the incremental adjustments to SD was applied to these models to evaluate their performance. Two parameters were adjusted: the data utilization rate and the proportion of the data that was subject to the SD adjustment because changing the SD of 100% of the input data would eliminate any relationship between the training input and test output. Therefore, the noise was injected into 10%, 20%, and 30% of the training input. Consumption time and prediction accuracy were used as indexes of performance. The equation for calculating the accuracy of these models is given below.

(7) $$\mathrm{Accuracy}=1\;-\;\bar{\left(\frac{\left|\mathrm{TestOutput}\;-\;\mathrm{TestTarget}\right|}{\mathrm{TestTarget}}\right)}\;\times \;100\%$$

Table 6 shows that the accuracy rate for the polynomial regression (PR) model decreased as the proportion of noise increased. However, in some cases, the accuracy increased significantly. There was almost no change in the extremely short time consumption. However, due to the low utilization of the overall data, the model was sensitive to the noise.

The SVM model is inclined to be sensitive to noise, so the accuracy dropped as expected with the increase in noise (Table 7). Moreover, as the random number generator was set as constant in MATLAB, even when noise increased significantly, the accuracy did not change much. Due to the addition of noise, the training time of all SVM models was extended by about one second. However, as the noise increased, the training time only varied slightly.

Table 8 shows that the ANN with K-fold cross-validation was not sensitive to the noise and showed great fault tolerance ability. As the ratio and amount of noise increased, the accuracy decreased a little and, in some cases, improved slightly. Moreover, the training time increased significantly when the injection ratio was 20% and varied a little in other conditions.

## 3. FEA Model of Non-Visible Defect in Plexiglass

### 3.1. Model of Plexiglass with Non-Visible Defect and FEA Set-Ups

**Figure 13:**
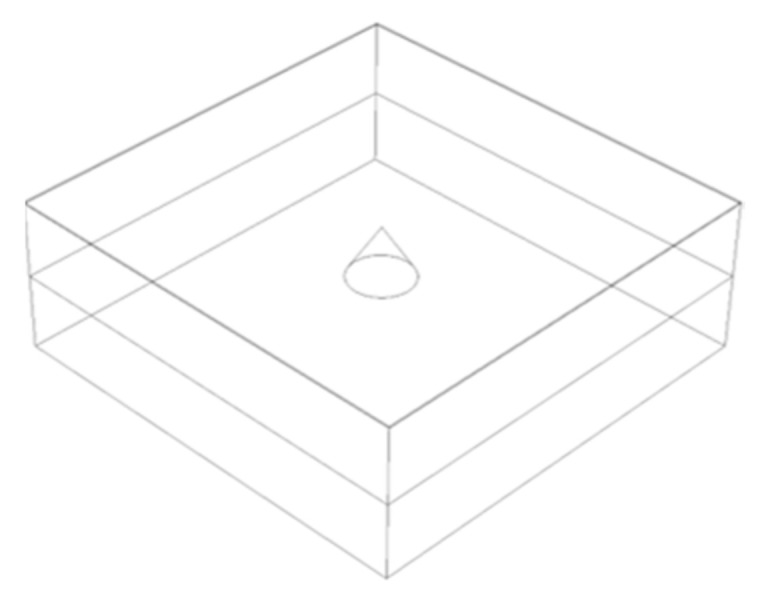
Perspective view of 3D model of experiment sample with non-visible defect.

The defects that exist in the plexiglass sample generally contain several different types, such as cracks on the surface or air bubbles beneath the surface, which could affect the quality of the products. Moreover, multiple defects in one plate could lead to difficulty in recognition. Hence, to improve defect detection accuracy, this research adopted a more specific condition, focusing on the plexiglass sample with a non-visible bubble defect. More specifically, this study was aimed to predict the diameter and depth of the bubble efficiently by using the data of the thermal imaging process. To conduct the FEA simulation, 3D models were constructed by using SolidWorks. Two plexiglass boards with the same dimensions (40 mm × 40 mm × 7 mm) were used. Between these two plates, one is intact, and the other one has a conical hole. To create the sample with a non-visible defect, the larger surface of these two boards was then overlapped, as shown in Figure 13. Moreover, considering the experimental conditions, the surface of the plexiglass sample could reflect lights from heating sources, which then affects the quality of IR images. Additionally, for the purpose of increasing the emissivity that is beneficial to thermal imaging results, a layer of black electrical tape (0.152 mm thickness) was stuck to the defect-free surface of the defective Plexiglass board. The upper surface of the tape is called the upper surface in subsequent studies. Since the target is to predict the bubble’s diameter and size, twelve models with bubbles of different sizes were built to accumulate the IR data. The diameters of bubbles are 1.98 mm, 2.38 mm, 4.37 mm, 5.95 mm, respectively. The depths of bubbles are 1.59 mm, 3.18 mm, 4.76 mm, respectively.

ANSYS Workbench was used to perform finite element analysis (FEA) of all models. To acquire temperature data through the whole process, the transient thermal condition was introduced. The ambient temperature was set as 298.15 K. To create the necessary thermal excitation, heat flux was applied to the upper surface of the models for the first 30 s. The amount of heat flux was set as 0.0012 W/mm^2^, which could provide the same heating power as two 90-w lamps. The upper surface was set under natural convection through the whole 150-s simulation process, while other surfaces were set as perfectly insulated to prevent heat loss. Moreover, thermal radiation loss from the upper surface to the environment was taken into consideration. For samples with non-visible defects, the distance between the two Plexiglass boards must be tiny enough to create the so-called artificial bubbles. Therefore, ‘Node Merge’ was applied to this contact region to eliminate the gap between boundaries. The thermophysical properties of plexiglass and air are listed in Table 9. Plexiglass and electrical tape’s density and thermal conductivity are much higher than these two for air (25 °C, 1 atm), while plexiglass has a higher heat capacity than the other two. Hence, the defect would result in differences in surface temperature distribution.

### 3.2. Outcomes and Analysis

**Figure 14:**
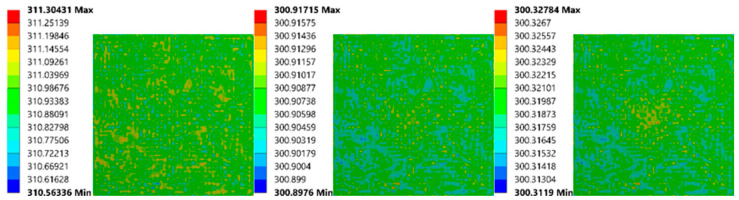
Simulation images of FEA model (1.98 mm diameter, 1.59 mm depth) at 30 s, 110 s, 150 s (from left to right).

To view the simulation outcomes intuitively, several thermal images of two models were exported from ANSYS. The first (as shown in Figure 14) has the smallest bubble (1.98 mm diameter, 1.59 mm depth). Since this bubble’s size is tiny, its impact on the upper surface was minimal, which did not appear until 110 s.

**Figure 15:**
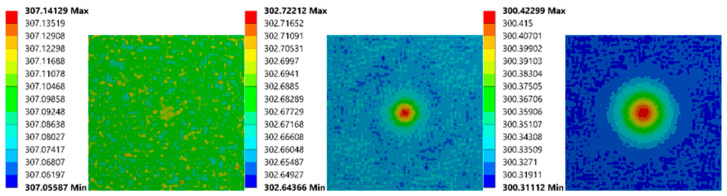
Simulation images of FEA model (5.95 mm diameter, 4.76 mm depth) at 33 s, 60 s, 150 s (from left to right).

The models with the biggest bubble outcomes (5.95 mm, 4.76 mm depth) are presented in Figure 15. The temperature difference first appeared at 33 s, which was near the end of the heating process. Besides that, from the color differences, it can be inferred that the value of the temperature differences is the most significant one.

**Figure 16:**
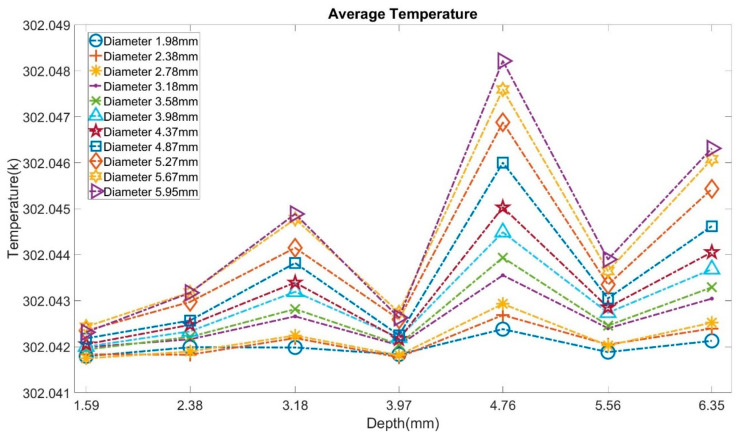
Average temperature through the cooling process from all models.

From the above models, it can be noticed that the defects with different sizes were not able to create observable differences on the upper surface through the heating process. While in the cooling process, the larger the bubble’s size, the faster the difference occurred, and the greater the area affected. However, the number of these FEA models is not enough to build a valid neural network model. Hence, more samples with extended diameters (2.78 mm, 3.18 mm, 3.58 mm, 3.98 mm, 4.87 mm, 5.27 mm, 5.67 mm) and depths (2.38 mm, 3.97 mm, 5.56 mm, 6.35 mm) were created. To analyze the potential trend more precisely, the average temperature of the upper surface through the cooling process is presented in Figure 16. In most cases, the model with the larger bubble outputs a higher temperature than the model with a smaller one. Therefore, the differences in the cooling process could be used to predict the bubble’s size.

### 3.3. Prediction Models for Diameter and Depth Prediction

To predict the diameter and depth of the bubbles found in the plexiglass boards, three neural network models were trained and compared by MATLAB. The first is a multi-input multi-output (MIMO) model that can simultaneously predict diameter and depth. The second and the last are multi-input single-output (MISO) models. The second was designed to predict diameter, while the last was used to predict depth.

The selection of input is the first parameter taken into consideration because it is critical to the efficiency of data preparation and prediction accuracy. The average temperature of the upper surface through each FEA model’s cooling process was decided as the input data for three reasons. The first is that each point’s temperature data on the upper surface through the process is difficult to export. The second is that the bubble’s diameter would be unknown before the prediction, so it is challenging to determine the appropriate input data area. The last is that the average temperature shows exploitative variation trends in the previous analysis. Besides that, in view of implementing the model in future experiments, it could be hard to maintain the ambient temperature as a constant. Therefore, the initial temperature was subtracted from the selected temperature data. The temperature was extracted every three seconds from the cooling process as input, which results in 40 numbers. Therefore, the number of nodes on input layers inside the network is 40. Otherwise, the number of input nodes could be too large to weaken the training efficiency. Additionally, these three models are single hidden layer structures.

Moreover, for general artificial neural network models, the number of training samples dramatically affects the model’s effectiveness and generalization ability. In the previous chapter, 77 sets of samples with non-visible bubbles that have combinations of different diameters and depths are built and simulated. Accordingly, the total number of training sets reached 77. To make full use of the temperature data of these limited models, K-fold cross-validation with R squared as the evaluation standard was utilized in these models, which has shown excellent performance in previous root depth prediction. The best number of nodes on the hidden layer was automatically selected in the training and testing of K-fold cv. Usually, the number of hidden neurons is set between the number of input and output nodes. For the MIMO model in this study, the range is from 3 to 39. For MISO models, it varies between 2 to 39. Apart from that, multi-core parallel operations and GPU acceleration were used to speed up the training process and avoid excessive calculation time.

To evaluate and compare these ANN models, 12 new samples with different combinations of diameter and depth were built and simulated under the same boundary conditions. The diameters are 2.18 mm, 3.38 mm, 4.57 mm, and 6.35 mm, respectively. The depths are 3.18 mm, 4.76 mm, and 6.35 mm, respectively. The accuracy of training and validation was calculated by Equation (7) For the validation accuracy of any single FEA model, it was calculated by the same equation without the ‘mean’ function.

#### 3.3.1. MIMO ANN Model

The MIMO neural network that could predict diameter and depth was trained at first. As listed in Table 10, the prediction accuracy of diameter and depth are both over 90%. Moreover, the accuracy of diameter prediction is higher than that of depth prediction. Besides that, the training costs almost half an hour because of the K-fold cv.

For diameter prediction, Table 11 shows that only two FEA models have less than 90% accuracy. Besides, the model with the smallest bubble has the lowest accuracy.

Table 12 shows a model with an accuracy that is less than 80% for depth prediction. Additionally, for models that have bubbles with the smallest diameters, the prediction accuracies are less than other models. Consequently, for most models, the MIMO ANN model’s predictions are exact. However, when the bubbles’ diameter is small, both diameter and depth prediction accuracies would decrease.

#### 3.3.2. MISO ANN Models

As shown in Table 13, the MISO ANN model for diameter prediction has a faster training speed and higher diameter prediction accuracy than those for the MIMO model.

As listed in Table 14, all models’ accuracy is higher than 90%, demonstrating that this neural network model provides convincing results on diameter prediction. It can be noticed that the models with the smallest depth tend to have comparatively lower accuracy than other models.

Table 15 displays that the MISO ANN model for depth prediction was also trained over 10% faster than the MIMO model. Moreover, the accuracy of depth prediction is higher than that of the MIMO model.

From Table 16, only one model has an accuracy that is less than 90%. More specifically, the MISO model only made a relatively inaccurate prediction on the FEA model with the smallest bubble.

After all, a MIMO ANN model could predict diameter and depth simultaneously and accurately. However, it took a little bit longer to train a MIMO than to train a MISO. Meanwhile, a MISO model for only predicting diameter or depth could result in more precise results. Although training two MISO models could take almost twice as long as training one MIMO model, it is still within an acceptable range.

### 3.4. Model Testing

**Figure 17:**
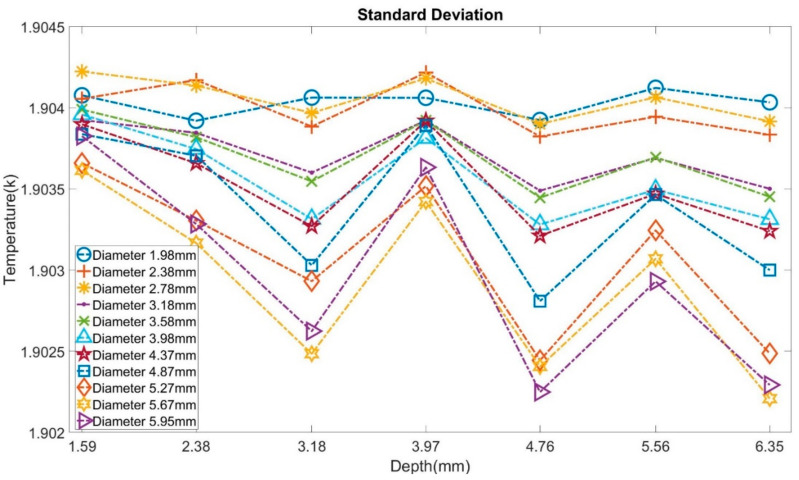
Standard deviation of temperature through the cooling process from all models.

The statistical tests based on the maneuvers of standard deviation were also introduced to verify these three ANN models’ fault-tolerant ability. At first, the standard deviation was analyzed to view the characteristics of temperature data used in neural network models, as shown in Figure 17. Models that have larger bubbles tend to output a lower standard deviation than models with smaller bubbles. In most cases, with the increase in diameter and depth, SD keeps decreasing.

Next, the statistical tests were applied. Increments of SD were set as 0.1, 0.2, and 0.3 according to the amount of original SD. Moreover, to keep the buried connection between the input and output, the proportions of data being adjusted were set as 10%, 20%, and 30%. The time consumption and validation accuracy are two indexes of evolution. The results of the MIMO model are given in Table 17. With the increase in noise, the computing time rises a lot. Moreover, in some cases, it even increases around 50%. However, the accuracy only slightly drops after the addition of noises and maintains over 85% for diameter and depth prediction.

The models for diameter prediction require a little more time to be trained after adjusting the SD, listed in Table 18. In most cases, the accuracies are above 90%. Only in two conditions, the accuracies drop significantly but are still higher than 85%.

Last is the model for depth prediction, and the results are shown in Table 19. The computing time increases after the addition of noise, it even increases over 60% in two cases, but the accuracy remains above 90%.

## 4. Conclusions and Future Directions

### 4.1. Conclusions

#### 4.1.1. RSA

Finite element analysis (FEA) was employed to simulate the process of applying infrared imaging technology to investigate plant root system architecture (RSA) below the soil. Based on preliminary findings that different root depth models will exhibit different surface temperature distributions, a line scan method was developed to predict the root structure. The main branches of the shallowly buried root (depth ≤ 5 mm, diameter = 1 mm) can be viewed from the prediction results, while the secondary roots were not to be located. Moreover, three dissimilar classification methods were applied to predict root depth (1–8 mm) and then compared. Two different polynomial regression sets were employed, reaching a 68.96% accuracy. In addition, three different data sets were established for Support Vector Machine (SVM) and Artificial Neural Network (ANN) model development. For SVM, cross-validation and grid search were used to improve performance. The SVM model was trained very quickly, and the accuracy was 86.80%, but the model was susceptible to noise. With K-fold cross-validation, the ANN required almost half an hour for training, but it had a better accuracy (87.97%) and fault-tolerance.

#### 4.1.2. Defect Detection

FEA has also been applied to simulate the IR imaging process of detecting non-visible bubbles (1.98–6.35 mm diameter, 1.59–6.35 mm depth) in plexiglass samples. As a result of the simulation, a bubble would lead to observable temperature differences on the upper surface during the cooling process. Moreover, as the bubble’s size increases, the upper surface’s average temperature keeps increasing. Based on this phenomenon, three ANN models were trained and compared. K-fold cv was also applied to improve the performance. The MIMO model could predict diameter and depth simultaneously. It reached 95.8% accuracy for diameter prediction and 91.7% for depth prediction. For the rest, two MISO models were both trained around 10% faster and achieved higher accuracy than the MIMO model. The diameter prediction model achieved 96.6% accuracy, and the other one for depth prediction outputted 95.4% accuracy. After all, statistical tests based on adjusting the SD were applied to these predictive models. All three ANN models consumed more time to be trained after adding the noise, but the accuracy was still around 90%, proving the tremendous fault-tolerant property.

### 4.2. Future Directions

Firstly, for the studies of the root system, the simulations can be conducted with different types of roots buried in dissimilar dry and wet mediums. Moreover, the current RSA model used for FEA can be further optimized to approach the real root system. For the detection of defects, more plexiglass samples with different size bubbles can be simulated to enrich the original data. Besides that, various heating and cooling settings can be tested to enlarge the differences between each sample.

Secondly, high-performance computing resources can be introduced to improve FEA models’ mesh size and method. Hence, the FEA models can produce more accurate results in a short period, which then provides better guidance for experiments. An IR camera with high resolution can also be applied to the experiments to acquire more accurate temperature readings.

Thirdly, efficient noise filtering methodologies are necessary for raw thermal images and temperature data, which would improve the accuracy of the line scan method for structure detection and machine learning models for depth prediction. To improve the performance and reduce the computing consumption, more suitable and effective parameter tuning techniques are required for ANN and SVM.

## Figures and Tables

**Table 1:** Constant thermophysical properties of materials.

Thermophysical Properties	Materials		
Materials Name	Soil [26]	Sugar beet Roots [27]	Acrylic Glass [28]
ρ (kg·m^−3^)	1300	1169.9	1150
k (W·m^−1^·K^−1^)	0.35	0.427	0.17
cp (J·K^−1^·kg^−1^)	830	3546.4	1470

**Table 2:** Average temperature and cooling rate of the selected point from each model.

Depth (mm)	1	2	3	4	4.5	5	6	7	8
Average Temperature (K)	296.86698	296.87029	296.87242	296.87437	296.87487	296.87544	296.87609	296.87647	296.87667
Cooling rate (K/s)	0.025674	0.025682	0.025682	0.025671	0.025667	0.025661	0.025652	0.025645	0.025641

**Table 3:** Correlation coefficient between root depths and average temperature of upper surface during the cooling process.

Time (s)	10	20	30	40	50	60	70	80	90	100	110	120
CC	0.62	0.67	0.73	0.79	0.85	0.89	0.93	0.96	0.98	0.99	0.99	0.98

**Table 4:** Results of SVM models.

Model Name	SVM		
Input Set	1st	2nd	3rd
Training accuracy (%)	68.15	70.70	66.31
Validation accuracy (%)	60.26	41.03	61.70

**Table 5:** Results of ANN models.

Model Name	ANN			ANN with K-Fold cv		
Input Set	1st	2nd	3rd	1st	2nd	3rd
Number of hidden layer’s neurons	10	10	10	30	5	16
Training accuracy (%)	85.13	73.42	82.93	92.98	91.24	92.70
Validation accuracy (%)	80.12	69.78	70.28	87.18	86.29	87.97

**Table 6:** Statistical tests of polynomial regression (PR) model.

Model	PR									
Noise Ratio (%)	0	10			20			30		
SD addition	0	0.02	0.04	0.06	0.02	0.04	0.06	0.02	0.04	0.06
Time (s)	0.007	0.001	0.001	0.001	0.001	0.001	0.001	0.001	0.001	0.001
Accuracy (%)	68.96	53.91	53.94	53.95	91.79	47.08	26.97	80.96	82.69	17.82

**Table 7:** Statistical tests of SVM model.

Model	SVM									
Noise Ratio (%)	0	10			20			30		
SD addition	0	0.02	0.04	0.06	0.02	0.04	0.06	0.02	0.04	0.06
Time (s)	9.57	10.03	11.35	10.17	10.27	10.10	11.35	10.20	10.09	10.16
Accuracy (%)	86.80	32.53	33.24	33.24	32.53	33.24	33.24	33.24	33.24	33.24

**Table 8:** Statistical tests of ANN with K-fold cross-validation model.

Model	ANN with K-Fold cv									
Noise Ratio (%)	0	10			20			30		
SD addition	0	0.02	0.04	0.06	0.02	0.04	0.06	0.02	0.04	0.06
Time (s)	1424	1424	1394	1448	1593	1597	1440	1568	1443	1433
Accuracy (%)	87.97	85.34	83.34	84.71	83.05	80.75	84.73	85.28	81.84	84.20

**Table 9:** Properties of materials.

Thermophysical Properties	Materials	
Materials Name	Plexiglass [29]	Air (25 °C, 1 atm) [30]
ρ (kg·m^−3^)	1185	1.184
k (W·m^−1^·K^−1^)	0.1934	0.02551
cp (J·K^−1^·kg^−1^)	1359	1007

**Table 10:** Overall statistics of MIMO ANN model.

Number of hidden layer’s neurons	31
Average Diameter training accuracy (%)	96.02
Average Depth training accuracy (%)	87.55
Average Diameter validation accuracy (%)	95.80
Average Depth validation accuracy (%)	91.67
Computing time (s)	1574.71

**Table 11:** Detailed validation accuracy of MIMO ANN model for diameter prediction.

Diameter Validation Accuracy (%)		Depth (mm)		
		3.18	4.76	6.35
Diameter (mm)	2.18	85.14	93.71	97.39
	3.38	95.64	99.73	98.26
	4.57	96.34	95.62	89.50
	5.35	98.62	99.86	99.82

**Table 12:** Detailed validation accuracy of MIMO ANN model for depth prediction.

Depth Validation Accuracy (%)		Depth (mm)		
		3.18	4.76	6.35
Diameter (mm)	2.18	84.06	76.10	82.44
	3.38	86.27	98.59	95.43
	4.57	98.51	89.82	94.32
	5.35	96.63	99.78	98.09

**Table 13:** Overall statistics of MISO ANN model for diameter prediction.

Number of hidden layer’s neurons	18
Average training accuracy (%)	96.91
Average validation accuracy (%)	96.63
Computing time (s)	1444.81

**Table 14:** Detailed validation accuracy of MISO ANN model for diameter prediction.

Diameter Validation Accuracy (%)		Depth (mm)		
		3.18	4.76	6.35
Diameter (mm)	2.18	93.88	95.36	96.05
	3.38	99.97	97.53	99.55
	4.57	99.44	98.93	93.04
	5.35	91.43	97.15	97.19

**Table 15:** Overall statistics of MISO ANN model for depth prediction.

Number of hidden layer’s neurons	3
Average training accuracy (%)	94.01
Average validation accuracy (%)	95.36
Computing time (s)	1372.61

**Table 16:** Detailed validation accuracy of MISO ANN model for depth prediction.

Depth Validation Accuracy (%)		Depth (mm)		
		3.18	4.76	6.35
Diameter (mm)	2.18	83.91	90.30	99.30
	3.38	96.85	96.00	97.04
	4.57	90.95	99.61	97.64
	5.35	96.72	98.17	97.80

**Table 17:** Statistical tests of MIMO ANN with K-fold cross-validation model.

Model	MIMO ANN with K-Fold cv									
Noise Ratio (%)	0	10			20			30		
SD addition	0	0.1	0.2	0.3	0.1	0.2	0.3	0.1	0.2	0.3
Time (s)	1575	1616	2431	1883	1846	1795	1902	2135	2226	2108
Diameter accuracy (%)	95.8	90.1	90.0	86.8	92.3	91.8	92.5	92.9	90.4	92.4
Depth accuracy (%)	91.7	87.6	89.7	88.8	85.4	88.8	89.2	88.7	90.7	88.3

**Table 18:** Statistical tests of MISO ANN with K-fold cv model for diameter prediction.

Model	MISO ANN with K-Fold cv for Diameter Prediction									
Noise Ratio (%)	0	10			20			30		
SD addition	0	0.1	0.2	0.3	0.1	0.2	0.3	0.1	0.2	0.3
Time (s)	1445	1647	1482	1576	1503	1525	1523	1515	1700	1555
Accuracy (%)	96.6	90.3	91.0	86.3	93.1	92.5	85.4	91.3	92.4	87.3

**Table 19:** Statistical tests of MISO ANN with K-fold cv model for depth prediction.

Model	MISO ANN with K-Fold cv for Depth Prediction									
Noise Ratio (%)	0	10			20			30		
SD addition	0	0.1	0.2	0.3	0.1	0.2	0.3	0.1	0.2	0.3
Time (s)	1373	1958	2267	1649	1736	1527	1660	1605	1541	2266
Accuracy (%)	95.4	93.8	90.2	91.2	93.4	90.0	91.6	92.8	91.1	90.4

## Data Availability

The data presented in this study are available on request from the corresponding author. The data are not publicly available due to future patent application.
